# A novel approach to identify the brain regions that best classify ADHD by means of EEG and deep learning

**DOI:** 10.1016/j.heliyon.2024.e26028

**Published:** 2024-02-09

**Authors:** Javier Sanchis, Sandra García-Ponsoda, Miguel A. Teruel, Juan Trujillo, Il-Yeol Song

**Affiliations:** a Lucentia Research Group - Department of Software and Computing Systems, University of Alicante, Carretera de San Vicente del Raspeig, s/n, San Vicente del Raspeig, 03690, Spain; b ValgrAI - Valencian Graduate School and Research Network of Artificial Intelligence, Camí de Vera s/n, 46022, Valencia, Spain; c Alicante Institute for Health and Biomedical Research (ISABIAL), Alicante, Spain; d College of Information Science and Technology, Drexel University, 3141 Chestnut Street, Philadelphia, USA

**Keywords:** Attention-deficit hyperactivity disorder, Brain regions, Electroencephalogram, Feature selection methods, Deep learning

## Abstract

**Objective:**

Attention-Deficit Hyperactivity Disorder (ADHD) is one of the most widespread neurodevelopmental disorders diagnosed in childhood. ADHD is diagnosed by following the guidelines of Diagnostic and Statistical Manual of Mental Disorders, Fifth Edition (DSM-5). According to DSM-5, ADHD has not yet identified a specific cause, and thus researchers continue to investigate this field. Therefore, the primary objective of this work is to present a study to find the subset of channels or brain regions that best classify ADHD vs Typically Developing children by means of Electroencephalograms (EEG).

**Methods:**

To achieve this goal, we present a novel approach to identify the brain regions that best classify ADHD using EEG and Deep Learning (DL). First, we perform a filtering and artefact removal process on the EEG signal. Then we generate different subsets of EEG channels depending on their location on the scalp (hemispheres, lobes, sets of lobes and single channels) and using backward and forward stepwise feature selection methods. Finally, we feed the DL neural network with each set, and compute the $f_{1}$-*score*.

**Results and conclusions:**

Based on the obtained results, the Frontal Lobe (FL) (0.8081 $f_{1}$-*score*) and the Left Hemisphere (LH) (0.8056 $f_{1}$-*score*) provide more significant information detecting individuals with ADHD, than using the entire set of EEG Channels (0.8067 $f_{1}$-*score*). However, when combining the Temporal, Parietal and Occipital Lobes (TL, PL, OL), better results (0.8097 $f_{1}$-*score*) were obtained compared with using only the FL and LH subsets. The best performance was obtained using Feature Selection Methods. In the case of the Backward Stepwise Feature Selection method, a combination of 14 EEG channels yielded a 0.8281 $f_{1}$-*score*. Similarly, using the Forward Stepwise Feature Selection method, a combination of 11 EEG channels yielded a 0.8271 $f_{1}$-*score*. These findings hold significant value for physicians in the quest to better understand the underlying causes of ADHD.

## Highlights

• In conjunction with Brain Regions, we apply Feature Selection Methods using EEG channels as features.
• We use a novel Deep Learning Convolutional Neural Network to perform all experiments. The raw EEG signal is used as input.
• Improved reliability with 3 x 10-fold cross-subject validation and data and code full available.
• ADHD best characterized by Left Hemisphere and Frontal Lobe in our database.
• By using Feature Selection Methods, we have found a combination of 11 channels that best characterizes ADHD.

## Graphical abstract

**Figure:**
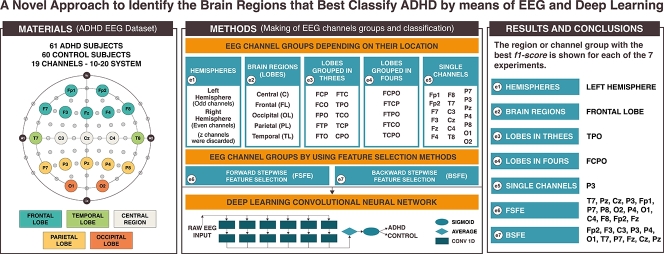

## 1 Introduction

Attention-Deficit/Hyperactivity Disorder (ADHD) is a prevalent neurodevelopmental disorder that significantly impacts children. It is characterized by symptoms such as inattention, impulsivity, and hyperactivity. In many cases, there are also associated sensory processing problems [1], that make the subject sensitive to physical stimuli via sound, sight, touch or smell and emotional stimuli. Therefore, early diagnosis and treatment can greatly enhance the quality of life of people who suffer this disorder, which is approximately a 7.6% of children, 5.6% of teenagers [2], and 6.76% of adults [3] worldwide.

Currently, ADHD diagnosis follows the Diagnostic and Statistical Manual of Mental Disorders, Fifth Edition (DSM-5). Besides DSM-5, more detailed, sophisticated assessment methods such as brain-based markers are used to enhance ADHD diagnosis accuracy and reliability [4], reducing subjectivity and biases. One of these markers is Electroencephalogram (EEG), which gathers the brain activity by means of small electrodes arranged over the scalp. EEG has several advantages compared with other brain-based markers such as Magnetoencephalography (MEG) [5], Magnetic Resonance Image (MRI) [6] and Positron Emission Tomography (PET) [7]; (i) EEG devices are more affordable, (ii) only requires a portable helmet with electrodes, (iii) it has a high temporal resolution, and (iv) it better tolerates subject's movements.

When working with ADHD and EEG, techniques such as Machine Learning (ML) [8], [9], [10], [11], [12], [13], [14], [15] and Deep Learning (DL) [16], [17], [18] are used. ML is a set of techniques comprising algorithms that automatically learn from data and develop optimized solutions. However, these techniques employ manual feature extraction, which is very time-consuming and presents difficulties in generalization, handcrafted features might be effective for specific datasets or tasks but may not generalize well to different populations or conditions. In contrast, DL is a powerful subset of ML that possesses the capability to address intricate problems by automatically discovering highly complex features. In addition, DL models can adapt to different EEG recording setups, electrode configurations, and experimental conditions with relatively minor adjustments. This adaptability is valuable in real-world scenarios where EEG data may vary. Finally, DL models can be trained incrementally, allowing them to adapt to new EEG data as it becomes available. This is crucial for applications where the EEG dataset grows over time, such as long-term monitoring of neurological conditions.

Despite the aforementioned advances, ADHD, as stated by the American Psychiatric Association [19], has not yet been attributed to a specific cause. Consequently, numerous studies are focused on uncovering its origins by studying the brain regions or channel subsets that best characterize ADHD. However, there is a divergence in the conclusions of these studies, highlighting the complexity of the issue.

Therefore, our main objective is to identify the brain regions or subsets of channels that best differentiate between TD and ADHD children using EEG signals. Pinpointing these crucial areas would offer significant benefits to both experts in the field and children with sensory processing problems. Furthermore, it can reduce the amount of EEG data processed by DL networks, thereby reducing training time and increasing data density, preserving the same informative content.

We have found articles with related objectives to ours such as [20], [21], [22], [23], that make a descriptive analysis of EEG signals from ADHD and TD, without applying more advanced methods of classification as ML or DL. We also found a set of studies [24], [15], [14] focused on the analysis of the connectivity among brain regions, applying only ML techniques. Other studies that go into more detail have found significant differences between ADHD and TD children in left hemisphere [25], [20], [21], in contrast to [26], [15], [27], who suggest that the right hemisphere can better distinguish between ADHD and TD controls. Regarding brain lobes, [20], [26], [23] find out that frontal lobe can classify better ADHD, [22] suggest that the posterior region does it better, while [15] conclude that occipital and temporal are the lobes that best classify ADHD. In [27] authors present a Machine Learning-Based Framework for the Classification of Children with ADHD and Healthy Controls. Additionally, they show the brain regions that best characterize ADHD. Nevertheless, we consider that there are many aspects to improve in that study: (i) it is said that the dataset is filtered but the source, [28], says it is unfiltered and to contain artifacts, (ii) it is not clear how they feed the algorithms, being that every input vector has a different length, (iii) it is not said how many subjects are taken to build the test and train sets, (iv) there is no mention of whether a cross-validation process has been carried out, and (v) the study exclusively utilizes machine learning algorithms with manual feature extraction. In [29], the researchers propose an explainable ML model for ADHD detection. Additionally, they study the brain regions that best characterize ADHD. They found that the Frontal Lobe is the brain region which best identifies ADHD. It should be noted that the authors don't specify whether data from the same subject are used in the training and test phases. In [30], the authors present optimal channel selection and features using statistical and ML techniques. In this study, they found a subset of channels along the entire scalp not attached to any brain region. As evident in the current literature, findings on the region that best identifies ADHD vary significantly. To the best of our knowledge, this challenge remains unsolved, warranting further research.

In order to solve the above problems, we present a novel methodology to Identify the Brain Regions that Best Classify ADHD by means of EEG and Deep Learning. Main contributions are: i) to the best of our knowledge, this study represents the first attempt to find the brain region or subset of channels by employing a DL model. By doing so, automatic feature extraction is performed, eliminating the need for manual feature engineering a time-consuming and error-prone process in traditional machine learning. Additionally, this approach enables further generalization to other EEG datasets related to ADHD. ii) In conjunction with Brain Regions, we apply Feature Selection Methods using EEG channels as features to find the subset of EEG channels that best characterize ADHD.

Finally, to present a more reliable and reproducible results, (i) we do use automatic pre-processing techniques to remove artifacts and noise, (ii) we apply a windowing process to ensure that all data have the same length, (iii) the training, validation and testing sets are created separating by subjects, (iv) a 10-fold cross-subject validation technique is conducted to make results less artificially optimistic and less biased, (v) since the data is not balanced, we use $f_{1}$-*score* to estimate the model's goodness, (vi) all experiments are conducted three times to mitigate the randomness of the DL model, (vii) results are contrasted by statistical tests, such as ANOVA or t-test, and (viii) this paper is fully reproducible, given that we use a public dataset, our code is available on *GitHub* [31], and we offer a comprehensive explanation of the whole process.

Eventually, this paper is organized as follows: Section 2 describes the EEG dataset and explains the methodology we used, Section 3 presents the experimental results and Section 4 discusses them. Section 5 shows the limitations of our work and Section 6 concludes this work.

## 2 Materials and methods

In this section, the dataset used in the experiments is described (sect. 2.1), as well as the applied methodology (sect. 2.2).

### 2.1 EEG dataset

**Figure 1:**
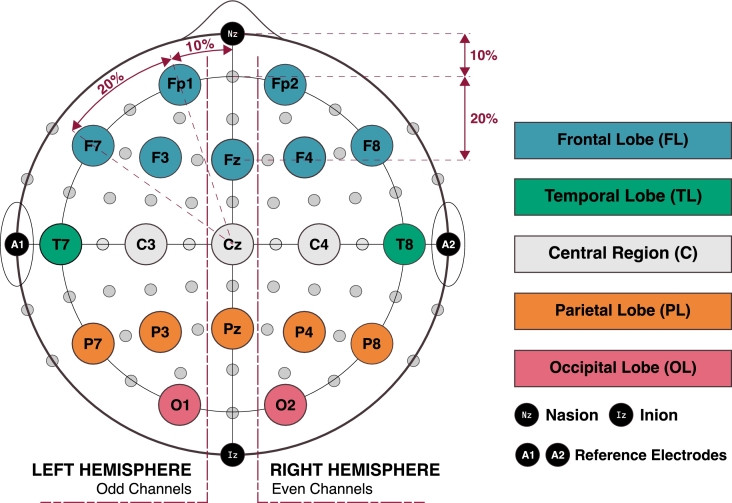
10-20 International electrode placement layout using 19 EEG channels. Created by authors with *Inkscape*.

The EEG Dataset used in this work has been obtained from [28]. In that dataset, there are 60 TD controls and 61 subjects suffering from ADHD aged between 7 and 12. All children were diagnosed by a psychiatrist according to the DSM-5 criteria. None of the TD controls had a history of psychiatric disorders or any report of high-risk behaviors. EEG recording was performed with a sampling frequency of 128Hz and based on the 10-20 electrode placement layout, using 19 channels [32], as can be seen in Fig. 1.

Patients were asked to count elements of a set of images. The number of items in each image varied from 5 to 16. To provide an uninterrupted stimulus during the signal recording, each image was shown forthwith after the child's response. Therefore, the duration of EEG recording for each patient depended on their performance. We have chosen 60 subjects for each group to have a balanced number of patients, removing one ADHD subject randomly.

As expected, the total number of samples from subjects suffering from ADHD is 924573 (equivalent to 1280.10 seconds, based on the sampling frequency of 128 Hz). These figures are higher than the 690055 total samples (equivalent to 5,391.02 seconds) recorded from TP Controls. Additionally, the mean Length of Recordings (LR) for subjects suffering from ADHD, denoted as $(LR_{adhd}=7,223.22$ seconds), was slightly higher than that for TP Controls, denoted as $(LR_{tp}=5,391.05$ seconds). Although the number of subjects is balanced, since the input to the DL model consists of time samples, it can be concluded that the dataset is unbalanced.

### 2.2 Methods

In this section, the methodology employed is explained. First, the EEG signal preprocessing is presented. Second, an explanation of the DL Model employed in this work is shown. Third, the evaluation metrics and the training process are also presented and eventually, we show how we look for the subset of EEG channels or brain regions that best classifies ADHD from TD controls.

#### 2.2.1 EEG signal preprocessing

Since the recording of EEG signals is very susceptible to noise, its highly recommended to perform a signal preprocessing prior to the classification step. In EEG, the noise and interferences are called artifacts i.e. eye blinks or heartbeats among others. To remove them, a three-step automatic signal preprocessing procedure has been performed by using the EEGLAB toolbox [33] and customized scripts.

First, we applied a 0.5 Hz high-pass FIR filter [34] in order to remove the signal drifting. Next, a 60 Hz low-pass FIR filter was also used. Since the highest frequency band in an EEG signal is gamma, γ(>30Hz), that can be divided in low-gamma (30-50 Hz) and high-gamma (50-100 Hz), by applying a 60 Hz low-pass filter, all the low-gamma information was retained and, at the same time, high frequency noise was removed. Eventually, we applied a 50 Hz Notch filter to attenuate the power supply interference.

Second, we reduce the artifacts on the EEG signal by using the Artifact Subspace Reconstruction (ASR) algorithm [35] available in the Matlab's EEGLAB plugin [36]. After that, we normalized the data by using z-score.

Third, the data is divided into 2-second windows with a 50% overlap, resulting in a total of 10,573 windows from the recorded EEG of TP controls and 14,236 windows from the recorded EEG of ADHD subjects. Since our model's input consists of these 2-second windows, we consider the dataset to be unbalanced. With a sampling frequency of sf=128Hz, each window contains 2×128=256 samples. Consequently, we generated a data matrix $M\in R^{C\times S}$, where C=19 denotes the number of EEG channels, and S=256 denotes the number of collected samples. According to [37], this windowing procedure has demonstrated improved classification results in deep learning-based EEG systems.

#### 2.2.2 Deep learning model

**Figure 2:**
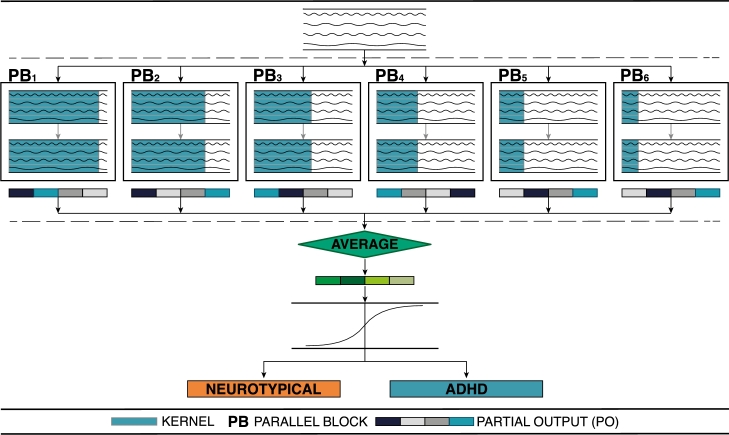
Multihead convolutional based neural network (EEG-MHCNet) architecture used in this work. Created by authors with *Inkscape*.

The DL model used in this work is a custom one, named EEG Multihead Convolutional Based Neural Network (EEG-MHCNet). The architecture of this model is illustrated in Fig. 2. We chose this model since, in a different study conducted towards the diagnosis of AHDH by using the same database as in this work [28], we obtained better $f_{1}$-*score* (0.8135) than state-of-art models like EEGNet (0.7771) [38] and VGGNet (0.7277) [39]. It also obtained better results than other architectures like MLP (0.7406).

The EEG-MHCNet is based on 1D Separable Convolutional Layers (SCL). A SCL is a Depthwise Convolution followed by a Pointwise Convolution [40]. The main advantages of SCL are that (i) they reduce the number of parameters to fit and (ii) they explicitly dissociate the relationship within and across feature maps by first learning a kernel summing up each feature map independently, and optimally merge the outputs afterwards. When applied to EEG signals, it learns features from each channel in time separately (Depthwise Convolution), then, it optimally combines the features obtained from each channel (Pointwise Convolution). Additionally, SCL have been used in the state-of-art deep learning EEG based model EEGNet obtaining excellent results in Brain-Computer Interfaces [38].

The design of the EEG-MHCNet model was based in the hypothesis of building a parallel architecture that could extract features from the input EEG signal at different frequencies, and the use of SCL to learn features for each EEG channel independently, optimally combining the extracted features from each EEG channel. Consequently, six Parallel Blocks (PB) were implemented.

Each PB consists of two-cascading SCL. For each PB, a different fixed kernel size was used. In the first parallel block ($PB_{1}$), the kernel length ($KL_{1}=64$) was initially set to half of the sampling frequency (sf/2=64). Subsequently, the kernel length was progressively halved for each consecutive parallel block until it reached a minimum of 2. This process resulted in the utilization of a total of 6 parallel blocks ($PB_{1}$ to $PB_{6}$). In addition, the output of each PB consists of a dense layer with a *softmax* activation. Finally, the partial output of each PB is averaged and classified by using a dense layer with *sigmoid* activation. Model hyperparameters, such as the number of filters in the SCL and the number of units in the dense layer at the end of the PB, were determined through an exhaustive search of various combinations. It's worth noting that the source code, which includes all hyperparameters, is available in [31].

#### 2.2.3 Input EEG channel groups

**Figure 3:**
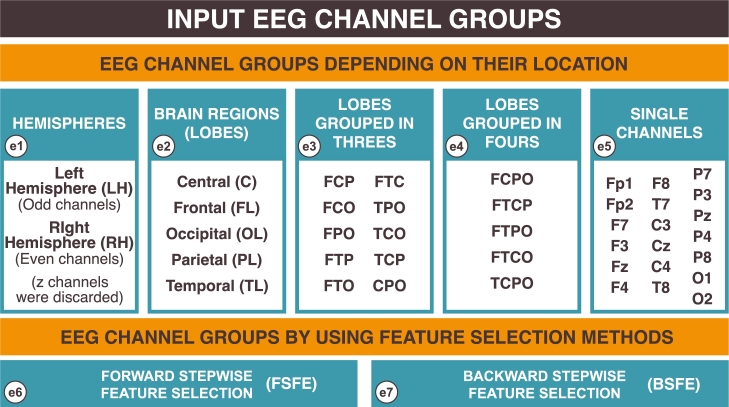
Input EEG channel groups. Created by authors with *Inkscape*.

The preprocessed EEG signals from 19 channels are taken as input features in individual and combinatorial sets to our DL classifier. The entire channel set is partitioned into several channel groups to find the subset that obtains the best results. All input EEG channel groups created are summarized in Fig. 3.

We can divide our conducted experiments into 2 groups (**G1**, **G2**); we have done five different experiments (**e1**, **e2**, **e3**, **e4** and **e5**) belonging to the first group and two experiments (**e6** and **e7**) that belong to the second group, as shown below:

**G1** Experiments ran by picking EEG channels depending on their location on the scalp:
  **e1** Channels according to the hemisphere that they belong to.
  **e2** Channels according to the brain lobe that they belong to.
  **e3** All possible combinations by grouping brain lobes in threes.
  **e4** All possible combinations by grouping brain lobes in fours.
  **e5** Every single channel as input to the model.
**G2** Experiments done picking EEG channels by using feature selection methods:
  **e6** Forward Stepwise Feature Selection Method (FSFS) [41] has been carried out by taking channels as variables for the model.
  **e7** Backward Stepwise Feature Selection Method (BSFS) [41] has been carried out by taking channels as variables for the model.

The first channels partition that we have made (**e1**) is related with hemispheres, so we divide channels into two groups: channels located in the right or left hemispherem, then a partition by brain lobes has been done (**e2**). Channels corresponding to each partition, (**e1**) and (**e2**), can be observed in the Table 1. We also use this brain lobes partition to make all possible combinations with three and four lobes (**e3** and **e4** respectively). The last separation of channels is made by using a single channel as input feature (**e5**). We do these partitions for the sake of knowing both whether one subset could distinguish better this disease than the other subsets and whether it is possible to obtain the same or better results with one subset than using all channels.

**Table 1:** Channel clustering by brain hemisphere and lobe.

Region	Abbreviation	Channels
*Left Hemisphere*	LH	Fp1, F3, F7, C3, T7, P3, P7, O1
*Right Hemisphere*	RH	Fp2, F4, F8, C4, T8, P4, P8, O2

*Central Region*	C	C3, Cz, C4
*Frontal Lobe*	FL	Fp1, Fp2, F7, F3, Fz, F4, F8
*Occipital Lobe*	OL	O1, O2
*Parietal Lobe*	PL	P7, P3, Pz, P4, P8
*Temporal Lobe*	TL	T7, T8

It is worth noting that to find the subset of channels that best classifies ADHD, it would be necessary to train the model with each and every subset existing in the total set of channels. There is a branch of Mathematics, called *Set Theory*, [42] that works with a set generated by all possible subsets of a given set *S*, including the empty set and *S* itself. This subset is called *Power Set* and it is denoted as P(S). Moreover, if *S* is a finite set, it is possible to calculate its cardinality, denoted as |P(S)|, which is the number of all elements in the set P(S). Thus, if the cardinality of *S* is |S|, the cardinality of P(S) is $|P(S)|=2^{\left|S\right|}$. In the present case, we want to know how many subsets of channels we can make, so our origin set will be the set formed by all channels, that we will call X={Fp1, Fp2, F7, F3, F4, F8, P7, P3, P4, P8, T7, T8, Fz, C3, Cz, C4, Pz, O1, O2 }, with |X|=19. Therefore, the Power Set of *X* set is P(X), that has a cardinality of $|P(X)|=2^{\left|19\right|}=524288$. Accordingly, we would need to train, validate and test 524287 different subsets (removing the empty subset) to assure that the best subset of channels we obtain is the subset that best classifies ADHD with our data and model. Training numerous subsets is impractical for us due to the extensive time and computational expenses involved. That is why there are other techniques that explore a far more restricted set of variables, such as *Stepwise Feature Selection Methods*.

In this study we have used *Forward Stepwise Feature Selection* (FSFS) and *Backward Stepwise Feature Selection* (BSFS) Methods [43]. FSFS begins by considering a set with no variables, and then adds variables one-at-a-time, until considering the model with all variables. In this case, the channels act as variables for the model. This process is outlined below:

1.1 Generate all possible models $M_{i}$ adding one variable/channel $V_{i}$:
  $M_{1}=\{V_{1}\},M_{2}=\{V_{2}\},\ldots ,M_{19}=\{V_{19}\}$
1.2 Evaluate each model $M_{i}$ giving a score $S_{i}$, which will be $f_{1}$-*score* in our case:
  $M_{1}\to S_{1},M_{2}\to S_{2},\ldots ,M_{19}\to S_{19}$
1.3 Choose the best score. In our case it will be the maximum score. Let us assume that the maximum in this example is $S_{2}$:
  $\max(S_{1},S_{2},\ldots ,S_{19})=S_{2}$
1.4 Select all variables from the model that has the best score:
2.1 Generate all models adding one of the remaining variables:
  $M_{1}=\{V_{2}+V_{1}\},M_{2}=\{V_{2}+V_{3}\},M_{3}=\{V_{2}+V_{4}\},\ldots ,M_{18}=\{V_{2}+V_{19}\}$
2.2 Evaluate each model giving a score:
  $M_{1}\to S_{1},M_{2}\to S_{2},\ldots ,M_{18}\to S_{18}$
2.3 Choose the best score. Let us assume that the maximum now is $S_{10}$:
  $\max(S_{1},S_{2},\ldots ,S_{18})=S_{10}$
2.4 Select all variables from the model that has the best score:
... Repeat these four steps until all variables are picked.
19.1 Generate all models adding one of the remaining variables:
  $M_{1}=\{V_{2}+V_{11}+\ldots +V_{5}\}$ (all variables)
19.2 Evaluate each model giving a score:
  $M_{1}\to S_{1}$
19.3 Choose the best score:
  $\max(S_{1})=S_{1}$
19.4 Select all variables from the model that has the best score:
  (all variables)

Thus, for each iteration *k*, with k∈{0,...,18}, we have to evaluate 19−k models, so eventually we need to evaluate $\sum_{k=0}^{18}(p-k)=p(p+1)/2=190$, which is much a smaller amount than the 524287 subsets of variables.

On the other hand, BSFS provides another alternative method to find the best subset of variables. Unlike FSFS, it begins evaluating the model with all variables and then, one-at-a-time, the algorithm removes the least useful variables until it remains with only one variable. Once again, we reduce drastically the number of models from 524287 to 190. We outline this process below:

1.1 Generate all possible models $M_{i}$ removing one variable $V_{i}$:
  $M_{1}=\{V_{2}+\ldots +V_{19}\},M_{2}=\{V_{1}+V_{3}+\ldots +V_{19}\},\ldots ,M_{19}=\{V_{1}+\ldots +V_{18}\}$
1.2 Evaluate each model $M_{i}$ giving a score $S_{i}$, which will be $f_{1}$-*score* in our case:
  $M_{1}\to S_{1},M_{2}\to S_{2},\ldots ,M_{19}\to S_{19}$
1.3 Choose the best score. In our case it will be the maximum score. Let us assume that the maximum in this example is $S_{1}$:
  $\max(S_{1},S_{2},\ldots ,S_{19})=S_{1}$
1.4 Select all variables from the model that has the best score:
2.1 Generate all models removing one of the remaining variables:
  $M_{1}=\{V_{3}+\ldots +V_{19}\},M_{2}=\{V_{2}+V_{4}+\ldots +V_{19}\},\ldots ,M_{19}=\{V_{2}+\ldots +V_{18}\}$
2.2 Evaluate each model giving a score:
  $M_{1}\to S_{1},M_{2}\to S_{2},\ldots ,M_{18}\to S_{18}$
2.3 Choose the best score. Let us assume that the maximum now is $S_{13}$:
  $\max(S_{1},S_{2},\ldots ,S_{18})=S_{13}$
2.4 Select all variables from the model that has the best score:
... Repeat these four steps until all variables are eliminated.
19.1 Generate all models removing one of the remaining variables:
  $M_{1}=\{V_{3}\}$
19.2 Evaluate each model giving a score:
  $M_{1}\to S_{1}$
19.3 Choose the best score:
  $\max(S_{1})=S_{1}$
19.4 Select all variables from the model that has the best score:

#### 2.2.4 Training process

In order to obtain reliable results, it is essential that the frames used for training, validation, and testing the model come from different subjects. As a result, we conducted a 10-fold cross-subject validation. Given the presence of 60 subjects in each group, during each iteration, we selected data from 54 ADHD-affected subjects and 54 TD individuals for training. Additionally, data from 6 ADHD subjects and 6 TD subjects were reserved for testing. Furthermore, from the training dataset, we chose 5 subjects from each class for validation. It should be noted that, in order to make these results reproducible, we applied a seed to the random algorithm responsible for selecting the subjects. As a result, the randomly selected subjects for each group remain consistent in each training turn. For the training process, the *ADAM* algorithm was used as optimization method [44]. In addition, the *Binary Cross Entropy* was employed as loss metric. These training parameters were chosen because we are dealing with a binary classification problem.

## 3 Results

In this section we present the results of our conducted experiments. We carried out seven experiments by combining different subsets of channels that act as input variables for our model. We have calculated four metrics for each model: $f_{1}$-*score*, accuracy, precision and recall [45]. To obtain more general and realistic results, all experiments were performed with a 10-fold inter-subject validation technique and each was repeated 3 times to mitigate the effects of random initialization of model weights. Therefore, we have 3×10=30 results for evaluation metrics per each model trained. The results shown in this section are the mean of those 30 iterations that we made for every model. Eventually, we compute the Confidence Interval (CI) with α=0.05 and perform an ANOVA test to know if the comparison among models is statistically significant or not.

To properly visualize these results, we present two graphs for each of the seven experiments conducted. First, a box plot to graphically represent the distribution of the 30 $f_{1}$-*scores* obtained by running 30 iterations of each subset, and second, the CI for estimating the $f_{1}$-*score* of each subset.

It is worth noting that we have compared all these results with those obtained by using all channels together, since one of the goals of this study is to know if it is possible to reduce the number of channels without removing relevant information. Hence, we gain insights into key brain regions for ADHD detection and enhance the comfort of children with heightened sensitivity.

### 3.1 First group results (G1)

The first group of experiments, **G1**, is composed by the experiments we ran based on the location of channels. We carried out five experiments: **e1** based on the hemispheres, **e2** conditioned by brain lobes, **e3** grouping brain lobes in threes, **e4** grouping brain lobes in fours and **e5** using only one channel.

**Figure 4:**
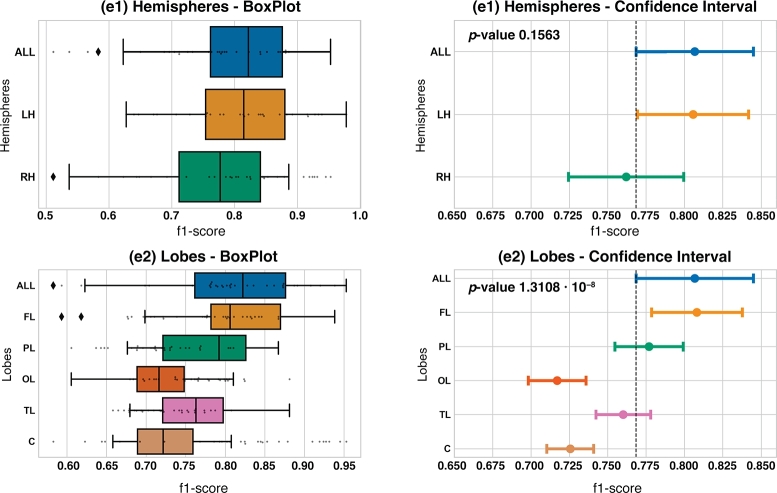
BoxPlots and confidence intervals related to **e1** brain hemispheres experiment and **e2** brain lobes experiment.

First, in Fig. 4 we show the results for the **e1** and **e2** experiments. Regarding **e1**, $f_{1}$-*score* metrics of ALL subset and Left Hemisphere channels (LH) subset are very similar, while $f_{1}$-*score* of Right Hemisphere channels (RH) subset is slightly smaller and it has a different distribution. However, we have obtained a p-value of 0.1563>0.05, which means that there are no statistically significant differences among $f_{1}$-*scores* of the subsets. See Table 2 for detailed results.

**Table 2:** Results table of experiment **e1**, brain hemispheres.

Region	f1-score	Accuracy	Precision	Recall
*All Channels*	0.8067	0.7609	0.7813	0.8551
*Left Hemisphere*	0.8056	0.7462	0.7565	0.8880
*Right Hemisphere*	0.7620	0.7039	0.7269	0.8274

Regarding **e2**, $f_{1}$-*score* metrics vary. We obtained a p-value of $1.3108\cdot 10^{-8}<0.05$, so that means that there are statistically significant differences among $f_{1}$-*scores* of the subsets. Specifically, it can be observed that there are no statistically significant differences among ALL, FL, PL and TL subsets regarding $f_{1}$-*score* of the subsets. However, we can see that OL and C subsets have it with ALL and FL subsets. Detailed results are shown Table 3.

**Table 3:** Results table of experiment **e2**, lobes.

Lobe	f1-score	Accuracy	Precision	Recall
*All Channels*	0.8067	0.7609	0.7813	0.8551

*Frontal Lobe*	0.8081	0.7494	0.7454	0.9015
*Parietal Lobe*	0.7769	0.7117	0.7106	0.8702
*Temporal Lobe*	0.7601	0.6699	0.6571	0.9088
*Central Region*	0.7255	0.6142	0.6136	0.8948
*Occipital Lobe*	0.7170	0.5996	0.6019	0.8938

**Figure 5:**
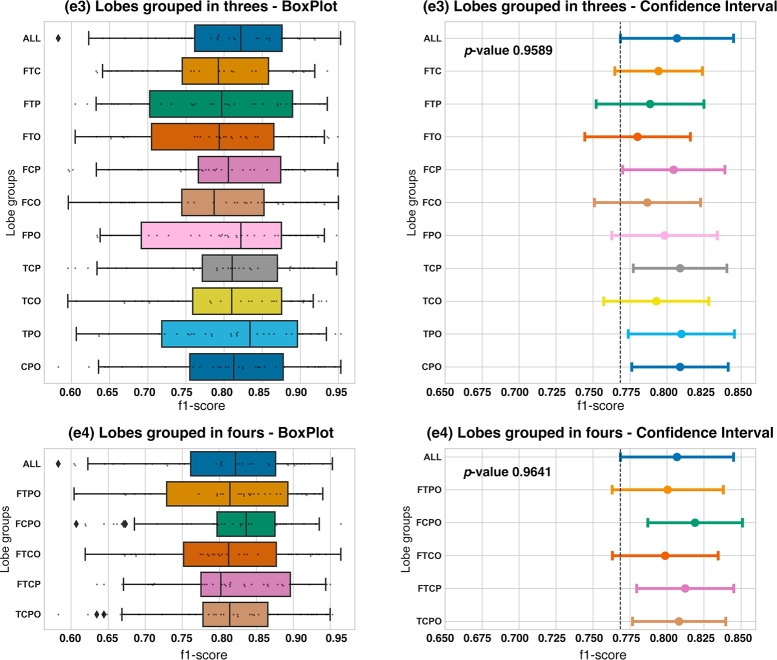
Boxplot and confidence interval with respect to **e3** and **e4**, lobes grouped into threes and fours experiments, respectively.

Second, in Fig. 5 we show the results for the **e3** grouping brain lobes in threes and **e4** grouping brain lobes in fours.

Regarding **e3** and **e4**, $f_{1}$-*score* metrics from all subsets have a similar mean value. The main difference among all subsets is the distribution of the samples; some have a wider and some have a shorter Interquartile Range (IQR), which is the difference between Q3 and Q1 quartiles. As we can foresee from the box plots, we obtained a p−value=0.9589>0.05 in **e3** and p−value=0.9641>0.05 in **e4**, which means that there are no statistically significant differences among $f_{1}$-*scores* of the subsets.

For a comprehensive understanding of the results in **e4** and **e5**, we invite you to consult both, Table 4 and Table 5. These tables provide a detailed breakdown of key performance metrics, including Accuracy, precision, recall, and F1-score.

**Table 4:** Results table of experiment **e3**, lobes grouped in threes.

Lobes groups	f1-score	Accuracy	Precision	Recall
*All Channels*	0.8067	0.7609	0.7813	0.8551

*TPO*	0.8097	0.7589	0.7635	0.8755
*TCP*	0.8088	0.7639	0.7767	0.8659
*CPO*	0.8088	0.7626	0.7738	0.8711
*FCP*	0.8045	0.7497	0.7624	0.8777
*FPO*	0.7983	0.7398	0.7487	0.8748
*FTC*	0.7943	0.7301	0.7442	0.8834
*TCO*	0.7927	0.7365	0.7513	0.8617
*FTP*	0.7885	0.7302	0.7472	0.8623
*FCO*	0.7867	0.7225	0.7427	0.8682
*FTO*	0.7800	0.7140	0.7298	0.8637

**Table 5:** Results table of experiment **e4**, lobes grouped in fours.

Lobes groups	f1-score	Accuracy	Precision	Recall
*All Channels*	0.8067	0.7609	0.7813	0.8551

FCPO	0.8190	0.7692	0.7822	0.8834
FTCP	0.8123	0.7601	0.7717	0.8817
TCPO	0.8081	0.7627	0.7843	0.8603
FTPO	0.8004	0.7429	0.7463	0.8790
FTCO	0.7988	0.7388	0.7573	0.8726

**Figure 6:**
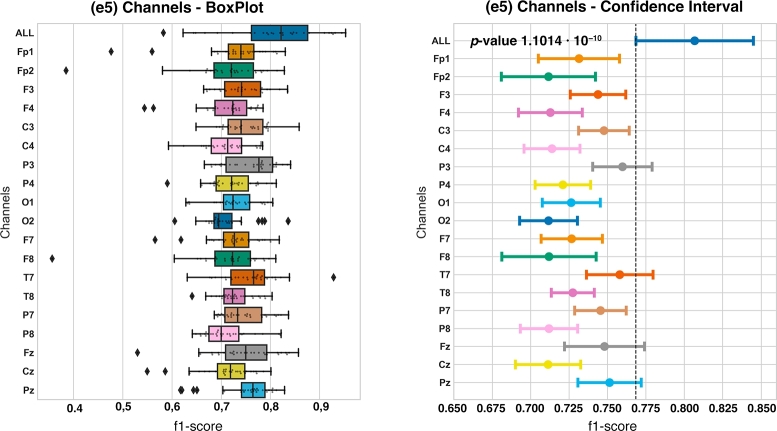
Boxplot and confidence interval with respect to the **e5** experiment.

The last experiment performed in this first group of experiments, **G1** is the **e5** using only one channel. Results can be observed in Fig. 6

Regarding **e5**, we can see in the box plot that $f_{1}$-*score* metrics from subsets with one channel seem different from the ALL subset. However, in the confidence interval, it can be seen that there are four subsets that have no statistically significant differences regarding the ALL subset, which are P3, T7, Fz and Pz subsets. Since we obtained a p-value of $1.1014\cdot 10^{-10}<0.05$, we can conclude that there are statistically significant differences among $f_{1}$-*scores* of the subsets. Some of these differences can be, for example, those we see when comparing the ALL subset with the fifteen subsets left (Fp1, Fp2, F3, F4, C3, C4, P4, O1, O2, F7, F8, T8, P7, P8 and Cz).

For a comprehensive analysis of our findings, including metrics such as Accuracy, precision, recall, and F1-score, please refer to the detailed results presented in Table 6.

**Table 6:** Results table of experiment **e5**, channels.

Channel	f1-score	Accuracy	Precision	Recall
*All Channels*	0.8067	0.7609	0.7813	0.8551

*T7*	0.7597	0.6731	0.6599	0.9064
*P3*	0.7580	0.6712	0.6621	0.8968
*Pz*	0.7513	0.6500	0.6338	0.9266
*Fz*	0.7480	0.6541	0.6423	0.9007
*C3*	0.7477	0.6438	0.6308	0.9231
*P7*	0.7454	0.6509	0.6442	0.8912
*F3*	0.7437	0.6262	0.6142	0.9492
*Fp1*	0.7314	0.6089	0.6022	0.9395
*T8*	0.7274	0.6101	0.6080	0.9138
*F7*	0.7266	0.5990	0.5954	0.9385
*O1*	0.7264	0.6086	0.6085	0.9102
*P4*	0.7209	0.5807	0.5827	0.9510
*C4*	0.7138	0.5715	0.5774	0.9408
*F4*	0.7128	0.5723	0.5778	0.9380
*P8*	0.7119	0.5741	0.5838	0.9223
*F8*	0.7118	0.5835	0.5852	0.9204
*O2*	0.7116	0.5705	0.5792	0.9315
*Fp2*	0.7115	0.5738	0.5787	0.9323
*Cz*	0.7112	0.5793	0.5839	0.9167

### 3.2 Second group results (G2)

**Figure 7:**
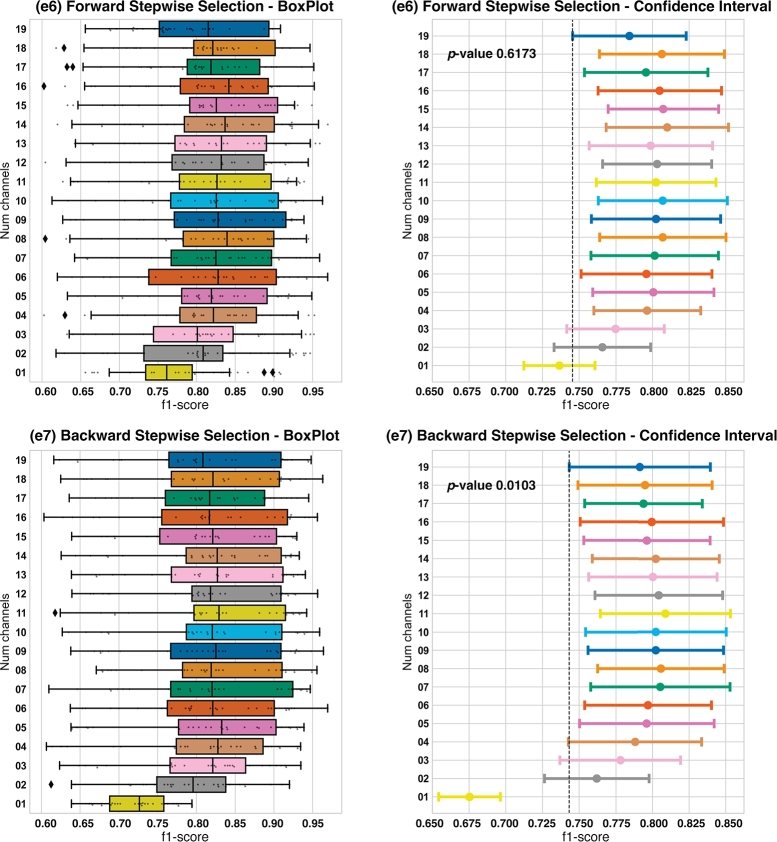
BoxPlots and confidence intervals related to **e6**, FSFS, and **e2**, BSFS.

This second group of experiments, **G2**, is composed by the experiments performed picking EEG channels by using feature selection methods. We carried out two experiments: **e6** using FSFS and **e7**, using BSFS. Results can be observed in Fig. 7.

Regarding **e6**, we compared 190 subsets, as we pointed out in section 2.2.3. The **e6** box plot of Fig. 7 shows the $f_{1}$-*score* metrics from all subsets. In this case, we can see that the increment in the number of channels is not directly proportional to the value of the $f_{1}$-*score*. It can be seen that the mean value of $f_{1}$-*score* for the subset with 19 channels is lower than the subset ran with 4 channels. However, after performing an ANOVA test, we obtained a p−value=0.6173>0.05, which means that there are no statistically significant differences among $f_{1}$-*scores* of the subsets.

For the Forward Stepwise Selection experiment **e6** results, we only present the results of the subset with best $f_{1}$-*score* for each number of channels, i.e., the best subset with one channel, the best subset with two channels, etc. Consequently, Table 7 has 19 rows. The corresponding channels are shown in Table 8. In this table, it is also observable which of the concrete channels are part of the subset.

**Table 7:** Results table of experiment **e6**, forward stepwise selection.

Num. Chans	f1-score	Accuracy	Precision	Recall
*All Channels*	0.8067	0.7609	0.7813	0.8551

*14*	0.8281	0.7857	0.7967	0.8832
*15*	0.8259	0.7797	0.7879	0.8886
*08*	0.8257	0.7725	0.7751	0.9024
*10*	0.8256	0.7741	0.7733	0.9022
*18*	0.8252	0.7785	0.7848	0.8875
*16*	0.8240	0.7751	0.7845	0.8891
*12*	0.8225	0.7787	0.7859	0.8817
*11*	0.8220	0.7722	0.7792	0.8925
*09*	0.8219	0.7748	0.7804	0.8854
*07*	0.8212	0.7695	0.7651	0.9020
*05*	0.8205	0.7720	0.7764	0.8888
*13*	0.8191	0.7711	0.7755	0.8864
*04*	0.8171	0.7686	0.7737	0.8885
*06*	0.8167	0.7604	0.7595	0.9003
*17*	0.8166	0.7677	0.7786	0.8782
*19*	0.8074	0.7554	0.7702	0.8741
*03*	0.7998	0.7396	0.7335	0.8934
*02*	0.7927	0.7263	0.7141	0.9021
*01*	0.7693	0.6818	0.6691	0.9150

**Table 8:** Forward stepwise selection channel description. It should be noted that performed with a 10-fold inter-subject validation technique and each was repeated 3 times to mitigate the effects of random initialization of model weights. Therefore, we have 3 × 10 = 30 results for evaluation metrics per each model trained. The results shown in this section are the mean of those 30 iterations that we made for every model.

Num. Chans.	Forward Selected Channels
*01*	T7
*02*	T7-Pz
*03*	T7-Pz-Cz
*04*	T7-Pz-Cz-P3
*05*	T7-Pz-Cz-P3-Fp1
*06*	T7-Pz-Cz-P3-Fp1-P7
*07*	T7-Pz-Cz-P3-Fp1-P7-P8
*08*	T7-Pz-Cz-P3-Fp1-P7-P8-O2
*09*	T7-Pz-Cz-P3-Fp1-P7-P8-O2-P4
*10*	T7-Pz-Cz-P3-Fp1-P7-P8-O2-P4-O1
*11*	T7-Pz-Cz-P3-Fp1-P7-P8-O2-P4-O1-C4
*12*	T7-Pz-Cz-P3-Fp1-P7-P8-O2-P4-O1-C4-F8
*13*	T7-Pz-Cz-P3-Fp1-P7-P8-O2-P4-O1-C4-F8-Fp2
*14*	T7-Pz-Cz-P3-Fp1-P7-P8-O2-P4-O1-C4-F8-Fp2-Fz
*15*	T7-Pz-Cz-P3-Fp1-P7-P8-O2-P4-O1-C4-F8-Fp2-Fz-C3
*16*	T7-Pz-Cz-P3-Fp1-P7-P8-O2-P4-O1-C4-F8-Fp2-Fz-C3-T8
*17*	T7-Pz-Cz-P3-Fp1-P7-P8-O2-P4-O1-C4-F8-Fp2-Fz-C3-T8-F3
*18*	T7-Pz-Cz-P3-Fp1-P7-P8-O2-P4-O1-C4-F8-Fp2-Fz-C3-T8-F3-F4
*19*	T7-Pz-Cz-P3-Fp1-P7-P8-O2-P4-O1-C4-F8-Fp2-Fz-C3-T8-F3-F4-F7

Regarding **e7**, where we compared 190 subsets, as we pointed out in section 2.2.3. The **e7** box plot of Fig. 7 shows the $f_{1}$-*score* metrics from all subsets. In this case, as well as in FSFS subsets, we can see that the increment in the number of channels is not directly proportional to the value of the $f_{1}$-*score*. It can be seen that the mean value of $f_{1}$-*score* for the subset with 19 channels is lower than the subset carried out with 5 channels. After performing an ANOVA test, we have obtained a p−value=0.0103<0.05, which means that there are statistically significant differences among $f_{1}$-*scores* of the subsets. Detailed results are shown in Table 9.

**Table 9:** Results table of experiment **e7**, backward stepwise selection.

Num. Channels	f1-score	Accuracy	Precision	Recall
*All Channels*	0.8067	0.7609	0.7813	0.8551

*11*	0.8271	0.7732	0.7716	0.9094
*08*	0.8246	0.7716	0.7671	0.9067
*07*	0.8243	0.7673	0.7641	0.9126
*12*	0.8235	0.7677	0.7655	0.9090
*14*	0.8219	0.7652	0.7665	0.9076
*10*	0.8218	0.7657	0.7681	0.9038
*09*	0.8218	0.7672	0.7673	0.9036
*13*	0.8202	0.7669	0.7697	0.8993
*16*	0.8197	0.7617	0.7649	0.9035
*06*	0.8176	0.7584	0.7532	0.9131
*15*	0.8170	0.7619	0.7669	0.8945
*05*	0.8169	0.7588	0.7562	0.9055
*18*	0.8160	0.7595	0.7639	0.8974
*17*	0.8151	0.7603	0.7672	0.8937
*19*	0.8131	0.7505	0.7529	0.9042
*04*	0.8105	0.7526	0.7533	0.8943
*03*	0.8025	0.7406	0.7390	0.8968
*02*	0.7896	0.7219	0.7154	0.8977
*01*	0.7202	0.5872	0.5883	0.9337

For the Backward Stepwise Selection experiment **e7**, we only present the results of the subset with best $f_{1}$-*score* for each number of channels. In this case the best subset with one channel, the best subset with two channels, and so on until we computed all channels. Consequently, Table 9 has 19 rows. The corresponding channels are shown in Table 10. In this table, it is also observable which of the concrete channels are part of the subset.

**Table 10:** Backward stepwise selection channel description. Discarded channels are in bold.

Num. Chans.	Backward Selected Channels
*19*	Fp1-Fp2-F3-F4-C3-C4-P3-P4-O1-O2-F7-F8-T7-**T8**-P7-P8-Fz-Cz-Pz
*18*	Fp1-Fp2-F3-F4-C3-C4-P3-P4-O1-O2-**F7**-F8-T7-P7-P8-Fz-Cz-Pz
*17*	Fp1-Fp2-F3-F4-C3-**C4**-P3-P4-O1-O2-F8-T7-P7-P8-Fz-Cz-Pz
*16*	Fp1-Fp2-F3-F4-C3-P3-P4-O1-O2-F8-T7-P7-**P8**-Fz-Cz-Pz
*15*	**Fp1**-Fp2-F3-F4-C3-P3-P4-O1-O2-F8-T7-P7-Fz-Cz-Pz
*14*	Fp2-F3-F4-C3-P3-P4-O1-**O2**-F8-T7-P7-Fz-Cz-Pz
*13*	Fp2-F3-**F4**-C3-P3-P4-O1-F8-T7-P7-Fz-Cz-Pz
*12*	Fp2-F3-C3-P3-P4-O1-**F8**-T7-P7-Fz-Cz-Pz
*11*	Fp2-F3-C3-P3-P4-O1-T7-P7-Fz-Cz-**Pz**
*10*	Fp2-F3-C3-**P3**-P4-O1-T7-P7-Fz-Cz
*09*	Fp2-**F3**-C3-P4-O1-T7-P7-Fz-Cz
*08*	Fp2-**C3**-P4-O1-T7-P7-Fz-Cz
*07*	Fp2-P4-O1-T7-**P7**-Fz-Cz
*06*	Fp2-P4-O1-**T7**-Fz-Cz
*05*	**Fp2**-P4-O1-Fz-Cz
*04*	P4-O1-**Fz**-Cz
*03*	P4-**O1**-Cz
*02*	P4-**Cz**
*01*	P4

### 3.3 Overall results

In order to provide a clear and concise overview of the results obtained from the seven experiments, we have compiled and presented them in Table 11.

**Table 11:** Sumarize results.

Experiment	f1-score	Accuracy	Precision	Recall
*e1*	0.8056	0.7462	0.7565	0.8880
*e2*	0.8081	0.7494	0.7454	0.9015
*e3*	0.8097	0.7589	0.7635	0.8755
*e4*	0.8190	0.7692	0.7822	0.8834
*e5*	0.7597	0.6731	0.6599	0.9064

*e6*	**0.8281**	0.7857	0.7967	0.8832
*e7*	0.8271	0.7732	0.7716	0.9094

## 4 Discussion

In this section we analyze the results shown in the previous section. We start by commenting on the findings of each experiment conducted and eventually, we discuss the most salient ones.

Looking at the results of first experiment, **e1**, we can say that there is no statistically significant differences among using the RH, the LH or all channels, so a reasonable option would be using the LH channels, given that this subset has a better CI than the RH one (Fig. 4) and it does not use all channels, only 8.

Regarding the brain lobes experiment **e2**, we can see that there are three brain regions (FL, PL and TL) with no statistically significant differences when using all channels (Fig. 4). However, the FL region provides a higher CI than the other regions and it gets the highest $f_{1}$-*score* mean for the **e2** experiment. Therefore, we could say that the best option from **e2** experiment would be using the FL channels. It is also worth noting that the FL subset is the largest subset, with 7 channels, so it is reasonable that this subset provides better results. On the contrary, the TL subset, with only two channels in it, is able to give the third better $f_{1}$-*score* of the **e2** experiment and could therefore be a feasible option if the reduction of the number of channels has to be prioritized.

In **e3**, joining lobes in threes experiment, the most interesting result is that there are no statistically significant differences between any of the subsets of channels. We can highlight the fact that there are three sets of channels that provide a higher $f_{1}$-*score* than the ALL subset: TPO, TCP and CPO. These three subsets have 9, 8 and 8 channels respectively, which is a considerable reduction of channels outperforming the $f_{1}$-*score* of the model fed with all channels. It is also worth noting that none of these three subsets has the FL in them, despite that the FL provided the best result in **e2** experiment. We also want to remark that the TCO combination, which is the combination with less channels (only 7) does not provide the worst result of the **e3** experiment.

Regarding the results obtained by grouping the lobes in fours in the **e4** experiment, we have once again no statistically significant differences between any of the subsets of channels. We have discovered three subsets with higher $f_{1}$-*score* mean than the ALL subset: FCPO, FTCP and TCPO. The first two subsets of channels have 17 channels and the third one has 12. This time, we do have the FL channels in two of the three combinations. We think that the TCPO option would be the best one of the **e4** experiment if we want to find a balance between $f_{1}$-*score* and as few channels as possible.

In **e5** experiment, we obtained the worst $f_{1}$-*scores* means because we only consider the data given by one channel to feed our model. Despite this, we were able to find 4 channels that have no statistically significant differences with the ALL subset: P3, T7, Fz and Pz (Fig. 6). If we relate these channels with their locations on the scalp (Fig. 1), we can see that none of these channels are placed in the right hemisphere. This may be because the information collected by the left hemisphere channels provides better insights for our model than that provided by the right hemisphere channels. In addition, another curious result can be seen joining **e3** and **e2** findings. We have said that the best brain lobe subset for our model was the FL, but in the **e3** experiment, none of the channels that provide better results are from the FL. That could be due to the fact that the FL channels do not have enough information on its own to feed a model properly, but joining them, they can provide powerful information for the model.

In the experiment **e6**, which was carried out by using FSFS method, there are no statistically significant differences between any subset of channels (Fig. 7), however, 16 out of 19 combinations of channels have higher $f_{1}$-*score* than the ALL subset (Table 7). In this case, the PL subset of channels seems to provide more profitable information than other brain regions, because it is the first region whose channels are all listed (combination with 9 channels in Table 8). Once again, we can see in Table 8 that the first channels selected by the algorithm came from the TL, PL and CL regions, and they all came from the LH. In turn, if we balance the amount of channels and the obtained $f_{1}$-*score*, the best option would be a combination with 8 channels (Fig. 7). This combination, as we can see in Table 8 consists of the following channels: T7, Pz, Cz, P3, Fp1, P7, P8 and O2.

Eventually, the experiment done using BSFS method, **e7**, has only one combination with statistically significant differences between the rest of the models (Fig. 7), which is the combination with only one channel. Since the BSFS removes the less useful channels for the model, we can say that the algorithm retains the most valuable channels. Exploring the Table 10, we can see that only 2 of the 11 channels that the algorithm removes last belongs to the RH (Pz, P3, F3, C3, P7, T7, **Fp2**, Fz, O1, Cz and **P4**). Therefore, this algorithm considers more important the LH channels and eliminates them last. In this experiment we can not clearly conclude anything about the brain lobes. However, if we had to choose the best results with the lowest number of channels, we will pick the combination with 7 channels: Fp2, P4, O1, T7, P7, Fz and Cz.

## 5 Limitations

Although we have achieved our goals in this work, namely finding the subsets of EEG channels that achieve the same or better performance in distinguishing ADHD subjects from TD controls as the total set of them, there are still some limitations. First, we only use one EEG-ADHD Dataset in this paper. In order to increase the reliability of this work, more EEG databases regarding ADHD should be tested with the same methodology. For example, this work could be extended by using [46], [47]. Second, regarding DL Neural Network explainability, it is a very known-problem that one of the main limitations of these models is the lack of interpretability [48]. Explaining why a DL model takes decisions is crucial in fields like medicine. Some eXplainable Artificial Intelligence (XAI) techniques like interpretable local surrogates, occlusion analysis, gradient-based techniques, and layerwise relevance propagation (LRP) [49] should be applied in future works in order to describe how the DL model used in this paper works. Third, we could try to increase the Accuracy and $f_{1}$-*score* by improving the DL model and/or performing a different preprocessing technique. By doing this, more accurate results could be presented.

## 6 Conclusions

Attention-Deficit Hyperactivity Disorder (ADHD) is one of the most common neurodevelopmental disorders diagnosed in childhood. ADHD is diagnosed following the guidelines of Diagnostic and Statistical Manual of Mental Disorders, Fifth Edition (DSM-5). According to DSM-5, ADHD has not yet a specific cause identified, thus scientists keep on researching on this area. In order to help researchers in finding the cause of ADHD, several works in finding the brain region or subset of channels that best distinguishes Typically Developing (TD) from ADHD children using EEG signals have been performed. However, the results obtained are very heterogeneous when revealing the depth of this problem. Thus, the main objective of this work is to present a novel approach to find the brain regions or subset of EEG channels that best classify ADHD vs TD children by using EEG as biomarker and a DL Neural Network as a classifier. As brain regions, we used Brain Hemispheres, Brain Lobes and combinations of Brain Lobes. As subset of channels, we used both a Forward and Backward Stepwise Feature Selection Methods (FSFS, BSFS) where the EEG channels were used as input features. According to the results obtained, we can conclude that in terms of brain regions, the Frontal Lobe (FL) (0.8081 $f_{1}$-*score*) and the Left Hemisphere (LH) (0.8056 $f_{1}$-*score*) provide the most information in the detection of ADHD subjects compared to the all EEG Channels Set (0.8067 $f_{1}$-*score*). However, the combination of the Temporal, Parietal and Occipital Lobes (TL, PL, OL) obtained better results (0.8097 $f_{1}$-*score*) than the FL and LH. Best performance was obtained by using Feature Selection Methods. In case of the FSFS, the combination of 11 EEG channels (T7, Pz, Cz, P3, Fp1, P7, P8, O2, P4, O1 and C4) obtained a 0.8271 $f_{1}$-*score*. In case of BSFS, the combination of 14 EEG channels (Fp2, F3, F4, C3, P3, P4, O1, O2, F8, T7, P7, Fz, Cz, and Pz) obtained a 0.8281 $f_{1}$-*score*. These findings may be useful to physicians and psychologists in further studies. As a future work, more ADHD EEG Datasets should be tested in order to improve the reliability of the obtained results. Since we are using a DL Neural Network, eXplainable Artificial Intelligence (XAI) techniques should be applied in future works in order to find out how IA obtains the results shown in this paper.

## CRediT authorship contribution statement

**Javier Sanchis:** Writing – review & editing, Writing – original draft, Validation, Software, Methodology, Investigation, Formal analysis, Data curation, Conceptualization. **Sandra García-Ponsoda:** Writing – original draft, Validation, Software, Methodology, Investigation, Formal analysis, Data curation, Conceptualization. **Miguel A. Teruel:** Writing – review & editing, Validation, Supervision, Software, Methodology, Investigation, Formal analysis, Data curation, Conceptualization. **Juan Trujillo:** Writing – review & editing, Validation, Supervision, Software, Methodology, Investigation, Funding acquisition, Formal analysis, Data curation, Conceptualization. **Il-Yeol Song:** Writing – review & editing, Validation, Supervision.

## Declaration of Competing Interest

The authors declare that they have no known competing financial interests or personal relationships that could have appeared to influence the work reported in this paper.

## Data Availability

Please note that all data and source code are publicly available. The source code repository is open access and can be reached as detailed in [31]. The data are accessible through [28]. Comprehensive instructions for installation and execution of the experiments are provided in the README.md file within the repository. The source code is implemented in Python and is designed to be run using the TensorFlow Docker environment, as specified in the GitLab repository.
